# (4-Acetyl­phenolato)(subphthalo­cyaninato)boron(III)

**DOI:** 10.1107/S1600536810046003

**Published:** 2010-11-20

**Authors:** Andrew S. Paton, Alan J. Lough, Timothy P. Bender

**Affiliations:** a Department of Chemical Engineering & Applied Chemistry, University of Toronto, 200 College Street, Toronto, Ontario, Canada M5S 3E5; b Department of Chemistry, University of Toronto, 80 St George Street, Toronto, Ontario, Canada M5S 3H6

## Abstract

In the title compound, C_32_H_19_BN_6_O_2_, the B atom adopts a BON_3_ tetra­hedral coordination geometry. In the crystal, pairs of mol­ecules are associated through aromatic π–π stacking inter­actions between the concave faces of the boronsubphthalocyanine fragments at a centroid–centroid distance of 3.4951 (19) Å and a weaker inter­action of the same type between the convex faces of the same group [centroid–centroid separation = 3.5669 (18) Å] also occurs.

## Related literature

For related structures and discussion of electronic effects, see: Paton *et al.* (2010 ▶). For further synthetic details, see: Claessens *et al.* (2002) ▶; Zyskowski & Kennedy (2000 ▶).
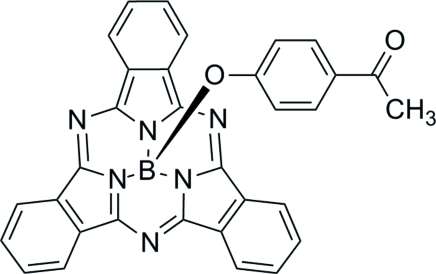


## Experimental

### 

#### Crystal data

C_32_H_19_BN_6_O_2_
*M* *_r_* = 530.34
Triclinic, 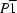

*a* = 10.5471 (8) Å
*b* = 10.5786 (5) Å
*c* = 11.5375 (9) Å
α = 77.446 (4)°
β = 88.817 (3)°
γ = 83.966 (4)°
*V* = 1249.54 (15) Å^3^
*Z* = 2
Mo *K*α radiation
μ = 0.09 mm^−1^
*T* = 150 K
0.08 × 0.08 × 0.05 mm

#### Data collection

Nonius KappaCCD diffractometer
Absorption correction: multi-scan (*SORTAV*; Blessing, 1995 ▶) *T*_min_ = 0.858, *T*_max_ = 1.002
8602 measured reflections
4273 independent reflections
2393 reflections with *I* > 2σ(*I*)
*R*_int_ = 0.078

#### Refinement

*R*[*F* ^2^ > 2σ(*F* ^2^)] = 0.055
*wR*(*F* ^2^) = 0.146
*S* = 0.98
4273 reflections
372 parameters
H-atom parameters constrained
Δρ_max_ = 0.23 e Å^−3^
Δρ_min_ = −0.22 e Å^−3^

### 

Data collection: *COLLECT* (Nonius, 2002 ▶); cell refinement: *DENZO-SMN* (Otwinowski & Minor, 1997 ▶); data reduction: *DENZO-SMN*; program(s) used to solve structure: *SIR92* (Altomare *et al.*, 1994 ▶); program(s) used to refine structure: *SHELXTL* (Sheldrick, 2008 ▶); molecular graphics: *PLATON* (Spek, 2009 ▶); software used to prepare material for publication: *SHELXTL*.

## Supplementary Material

Crystal structure: contains datablocks global, I. DOI: 10.1107/S1600536810046003/hb5719sup1.cif

Structure factors: contains datablocks I. DOI: 10.1107/S1600536810046003/hb5719Isup2.hkl

Additional supplementary materials:  crystallographic information; 3D view; checkCIF report

## Figures and Tables

**Table 1:** Selected bond lengths (Å)

B1—O1	1.457 (4)
B1—N1	1.487 (4)
B1—N3	1.494 (4)
B1—N5	1.487 (4)

**Fig. 1.**
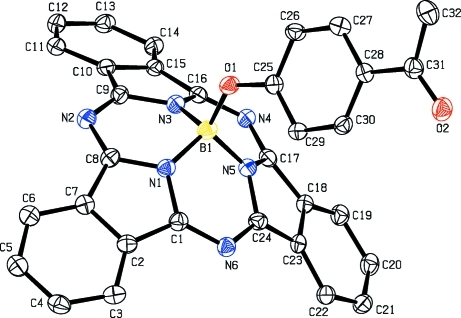
The molecular structure of AcPhO-BsubPc with displacement ellipsoids drawn at the 30% probability level.

**Fig. 2.**
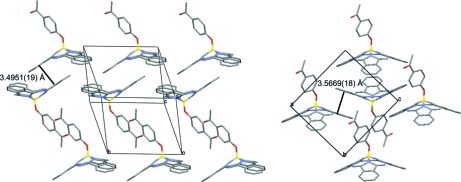
Extended crystal structure of AcPhO-BsubPc shown from two perspectives (side and end).

## supplementary crystallographic information

### Comment

We report the crystal structure of 4-acetylphenoxy-boronsubphthalocyanine (AcPhO-BsubPc), which possesses an electron withdrawing group in the *para* position of the phenoxy molecular fragment. We have recently reported a study of the crystal structures of a series of *para*-substituted-phenoxy-BsubPcs in which most of the substituents were alkyl (electron donating) (Paton *et al.*, 2010). Contained within the study was 4-fluorophenoxy-BsubPc (FPhO-BsubPc). While fluorine is moderately electron withdrawing, we did not observe any difference in its crystal structure compared to the baseline phenoxy-BsubPc. Our goal is to study the effect of the placement of strong electron withdrawing groups on the phenoxy molecular fragment and to determine any effect on the crystal structure of the resulting phenoxy-BsubPc. The structure of the current report is described and compared to FPhO-BsubPc, which typifies the phenoxy-BsubPc packing motif. The title compound was prepared by a method described previously (Paton *et al.*, 2010, Claessens *et al.*, 2002), in which chloro-boronsubphthalocyanine (Cl-BsubPc) is reacted with an excess of 4-hydroxy-acetophenone (4-acetylphenol) until substitution is complete. After purification, single crystals suitable for diffraction were grown using vapour diffusion of heptane into a solution of the product in benzene. The molecular structure of the title compound is shown in Fig. 1. The compound shows the expected bowl shape of the BsubPc ligand. The boron-oxygen-carbon (B—O—C) angle in the molecule is 130.3 (2)°; this value differs from both the experimental and computational gas-phase values of B—O—C angle for the typical FPhO-BsubPc, which are significantly smaller, at 115.2 (2)° and around 115°, respectively (Paton *et al.*, 2010). Looking at the torsion angle between the boron, oxygen, and the first two carbon atoms on the phenoxy substituent (B—O—C—C) gives a value of -22.0 (5). In contrast, the angle associated with typical phenoxy-BsubPc is -91.0 (2)° relative to the plane of the BsubPc fragment (Paton *et al.*, 2010). The extended crystal structure of AcPhO-BsubPc (Fig. 2), is typical to that which we have seen for *para*-alkylphenoxy-BsubPc when the alkyl group was sufficiently large (Paton *et al.* 2010). Each BsubPc molecular fragment associates with its nearest neighbour through a π-interaction [C18/C19/C20/C21/C22/C23 and C17/C18/C23/C24/N5, (-*x*, -*y*, -*z*)] between concave faces, with a centroid-to-centroid distance of 3.4951 (19) Å. The arrangement of the nearest neighbours is one-dimensional through the crystal parallel to the *b* axis of the unit cell and resembles something between the dimer arrangement seen for *p*-H, *p*-methyl and *p*-t-butylphenoxy-BsubPc and the ribbon arrangement seen for *p*-t-octylphenoxy-BsubPc. (Paton *et al.* 2010) Finally, there is a π-interaction linking adjacent rows between the BsubPc convex faces [C10/C11/C12/C13/C14/C15 and C9/C10/C15/C16/N3, (-*x*, -*y*, -*z*)] at a distance of 3.5669 (18) Å (Fig. 2).

### Experimental

Cl-BsubPc, synthesized by a procedure adapted from Zyskowski and Kennedy (2000). The title compound was synthesized using a method adapted from Claessens *et al.* (2002) and Paton *et al.* (2010): 4-Acetylphenoxy-boronsubphthalocyanine. Cl-BsubPc (0.510 g, 0.0012 mol) was mixed with 4-hydroxy-acetophenone (4-acetyl-phenol, 0.860 g, 0.0063 mol) in 1,2-dichlorobenzene (10 ml) in a cylindrical vessel fitted with a reflux condenser and argon inlet. The mixture was stirred and heated at reflux under a constant pressure of argon for 17 h. Reaction was determined complete *via* HPLC by the absence of Cl-BsubPc. The solvent was evaporated under rotary evaporation. The crude product purified on a Kauffman column using standard basic alumina (300 mesh) as the adsorbent and dichloromethane as the eluent. The product elutes from the Kauffman column while the excess phenol remains adsorbed. The dichloromethane was then removed under reduced pressure yielding a dark pink/magenta powder of the title compound (0.215 g, 34%).  For further details of the Kauffman apparatus, see: Paton *et al.* (2010).

### Crystal data

**Table:**

C_32_H_19_BN_6_O_2_	*Z* = 2
*M**_r_* = 530.34	*F*(000) = 548
Triclinic, *P*1	*D*_x_ = 1.410 Mg m^−^^3^
Hall symbol: -P 1	Mo *K*α radiation, λ = 0.71073 Å
*a* = 10.5471 (8) Å	Cell parameters from 8602 reflections
*b* = 10.5786 (5) Å	θ = 2.6–25.0°
*c* = 11.5375 (9) Å	µ = 0.09 mm^−^^1^
α = 77.446 (4)°	*T* = 150 K
β = 88.817 (3)°	Block, magenta
γ = 83.966 (4)°	0.08 × 0.08 × 0.05 mm
*V* = 1249.54 (15) Å^3^	

### Data collection

**Table:**

Nonius KappaCCD diffractometer	4273 independent reflections
Radiation source: fine-focus sealed tube	2393 reflections with *I* > 2σ(*I*)
graphite	*R*_int_ = 0.078
Detector resolution: 9 pixels mm^-1^	θ_max_ = 25.0°, θ_min_ = 2.6°
φ scans and ω scans with κ offsets	*h* = −12→12
Absorption correction: multi-scan from symmetry-related measurements (*SORTAV*; Blessing, 1995)	*k* = −11→12
*T*_min_ = 0.858, *T*_max_ = 1.002	*l* = −12→13
8602 measured reflections	

### Refinement

**Table:**

Refinement on *F*^2^	Secondary atom site location: difference Fourier map
Least-squares matrix: full	Hydrogen site location: inferred from neighbouring sites
*R*[*F*^2^ > 2σ(*F*^2^)] = 0.055	H-atom parameters constrained
*wR*(*F*^2^) = 0.146	*w* = 1/[σ^2^(*F*_o_^2^) + (0.0559*P*)^2^] where *P* = (*F*_o_^2^ + 2*F*_c_^2^)/3
*S* = 0.98	(Δ/σ)_max_ < 0.001
4273 reflections	Δρ_max_ = 0.23 e Å^−^^3^
372 parameters	Δρ_min_ = −0.22 e Å^−^^3^
0 restraints	Extinction correction: *SHELXTL* (Version 6.1; Sheldrick, 2008), Fc^*^=kFc[1+0.001xFc^2^λ^3^/sin(2θ)]^-1/4^
Primary atom site location: structure-invariant direct methods	Extinction coefficient: 0.0102 (19)

### Special details

**Table:**

Experimental. (SORTAV; Blessing, 1995)
Geometry. All e.s.d.'s (except the e.s.d. in the dihedral angle between two l.s. planes) are estimated using the full covariance matrix. The cell e.s.d.'s are taken into account individually in the estimation of e.s.d.'s in distances, angles and torsion angles; correlations between e.s.d.'s in cell parameters are only used when they are defined by crystal symmetry. An approximate (isotropic) treatment of cell e.s.d.'s is used for estimating e.s.d.'s involving l.s. planes.
Refinement. Refinement of *F*^2^ against ALL reflections. The weighted *R*-factor *wR* and goodness of fit *S* are based on *F*^2^, conventional *R*-factors *R* are based on *F*, with *F* set to zero for negative *F*^2^. The threshold expression of *F*^2^ > σ(*F*^2^) is used only for calculating *R*-factors(gt) *etc*. and is not relevant to the choice of reflections for refinement. *R*-factors based on *F*^2^ are statistically about twice as large as those based on *F*, and *R*- factors based on ALL data will be even larger.

### Fractional atomic coordinates and isotropic or equivalent isotropic displacement parameters (Å^2^)

**Table:**

	*x*	*y*	*z*	*U*_iso_*/*U*_eq_	
O1	0.84545 (19)	0.21044 (17)	0.32764 (17)	0.0390 (6)	
O2	1.2585 (2)	0.5988 (2)	0.3568 (2)	0.0493 (6)	
N1	0.7983 (2)	0.1720 (2)	0.1263 (2)	0.0334 (6)	
N2	0.7054 (2)	−0.0271 (2)	0.1944 (2)	0.0373 (6)	
N3	0.6430 (2)	0.1576 (2)	0.2758 (2)	0.0342 (6)	
N4	0.5094 (2)	0.3473 (2)	0.2935 (2)	0.0360 (6)	
N5	0.6990 (2)	0.3625 (2)	0.1770 (2)	0.0338 (6)	
N6	0.8102 (2)	0.3770 (2)	−0.0067 (2)	0.0356 (6)	
C1	0.8329 (3)	0.2456 (3)	0.0194 (3)	0.0348 (8)	
C2	0.8656 (3)	0.1542 (3)	−0.0569 (3)	0.0351 (8)	
C3	0.9125 (3)	0.1700 (3)	−0.1722 (3)	0.0416 (8)	
H3A	0.9329	0.2524	−0.2155	0.050*	
C4	0.9285 (3)	0.0610 (3)	−0.2218 (3)	0.0448 (9)	
H4A	0.9639	0.0685	−0.2990	0.054*	
C5	0.8939 (3)	−0.0592 (3)	−0.1609 (3)	0.0429 (8)	
H5A	0.9039	−0.1313	−0.1983	0.052*	
C6	0.8452 (3)	−0.0755 (3)	−0.0474 (3)	0.0385 (8)	
H6A	0.8211	−0.1574	−0.0064	0.046*	
C7	0.8325 (3)	0.0317 (3)	0.0055 (3)	0.0343 (8)	
C8	0.7819 (3)	0.0472 (3)	0.1197 (3)	0.0348 (8)	
C9	0.6314 (3)	0.0325 (3)	0.2668 (3)	0.0335 (7)	
C10	0.5121 (3)	0.0000 (3)	0.3263 (2)	0.0332 (8)	
C11	0.4524 (3)	−0.1143 (3)	0.3485 (3)	0.0366 (8)	
H11A	0.4909	−0.1905	0.3256	0.044*	
C12	0.3356 (3)	−0.1134 (3)	0.4047 (3)	0.0396 (8)	
H12A	0.2946	−0.1911	0.4235	0.048*	
C13	0.2768 (3)	−0.0006 (3)	0.4345 (3)	0.0389 (8)	
H13A	0.1966	−0.0030	0.4735	0.047*	
C14	0.3323 (3)	0.1145 (3)	0.4087 (3)	0.0368 (8)	
H14A	0.2901	0.1917	0.4268	0.044*	
C15	0.4514 (3)	0.1145 (3)	0.3557 (2)	0.0320 (7)	
C16	0.5348 (3)	0.2169 (3)	0.3156 (3)	0.0331 (7)	
C17	0.5876 (3)	0.4175 (3)	0.2165 (3)	0.0340 (8)	
C18	0.5647 (3)	0.5469 (3)	0.1419 (3)	0.0341 (8)	
C19	0.4719 (3)	0.6503 (3)	0.1443 (3)	0.0396 (8)	
H19A	0.4082	0.6439	0.2041	0.048*	
C20	0.4753 (3)	0.7628 (3)	0.0569 (3)	0.0406 (8)	
H20A	0.4158	0.8361	0.0594	0.049*	
C21	0.5650 (3)	0.7704 (3)	−0.0351 (3)	0.0404 (8)	
H21A	0.5648	0.8488	−0.0938	0.048*	
C22	0.6538 (3)	0.6665 (3)	−0.0425 (3)	0.0367 (8)	
H22A	0.7112	0.6703	−0.1074	0.044*	
C23	0.6558 (3)	0.5557 (3)	0.0492 (3)	0.0329 (7)	
C24	0.7357 (3)	0.4319 (3)	0.0686 (3)	0.0338 (7)	
C25	0.9382 (3)	0.2846 (3)	0.3453 (3)	0.0343 (8)	
C26	0.9781 (3)	0.2682 (3)	0.4620 (3)	0.0372 (8)	
H26A	0.9395	0.2093	0.5232	0.045*	
C27	1.0731 (3)	0.3366 (3)	0.4899 (3)	0.0398 (8)	
H27A	1.1005	0.3232	0.5700	0.048*	
C28	1.1292 (3)	0.4251 (3)	0.4021 (3)	0.0338 (7)	
C29	1.0886 (3)	0.4412 (3)	0.2854 (3)	0.0361 (8)	
H29A	1.1262	0.5014	0.2245	0.043*	
C30	0.9946 (3)	0.3714 (3)	0.2561 (3)	0.0366 (8)	
H30A	0.9689	0.3828	0.1758	0.044*	
C31	1.2310 (3)	0.5024 (3)	0.4297 (3)	0.0406 (8)	
C32	1.2969 (4)	0.4619 (3)	0.5468 (3)	0.0601 (10)	
H32A	1.3636	0.5190	0.5500	0.090*	
H32B	1.3355	0.3718	0.5572	0.090*	
H32C	1.2351	0.4684	0.6104	0.090*	
B1	0.7555 (3)	0.2280 (3)	0.2299 (3)	0.0362 (9)	

### Atomic displacement parameters (Å^2^)

**Table:**

	*U*^11^	*U*^22^	*U*^33^	*U*^12^	*U*^13^	*U*^23^
O1	0.0413 (13)	0.0386 (12)	0.0360 (13)	−0.0134 (10)	−0.0056 (10)	−0.0005 (10)
O2	0.0517 (15)	0.0442 (13)	0.0549 (16)	−0.0146 (11)	0.0061 (12)	−0.0127 (12)
N1	0.0343 (15)	0.0325 (14)	0.0330 (16)	−0.0058 (11)	0.0030 (12)	−0.0054 (12)
N2	0.0372 (16)	0.0352 (14)	0.0393 (16)	−0.0060 (12)	0.0015 (13)	−0.0068 (12)
N3	0.0364 (16)	0.0334 (14)	0.0334 (15)	−0.0067 (12)	0.0020 (12)	−0.0071 (12)
N4	0.0418 (16)	0.0333 (15)	0.0335 (16)	−0.0085 (12)	0.0027 (13)	−0.0067 (12)
N5	0.0368 (16)	0.0335 (14)	0.0318 (16)	−0.0063 (12)	0.0009 (12)	−0.0074 (12)
N6	0.0357 (16)	0.0345 (14)	0.0376 (16)	−0.0065 (12)	0.0006 (13)	−0.0083 (12)
C1	0.0325 (18)	0.0363 (18)	0.0355 (19)	−0.0065 (14)	0.0010 (15)	−0.0062 (15)
C2	0.0315 (18)	0.0375 (18)	0.035 (2)	−0.0002 (14)	−0.0030 (15)	−0.0053 (15)
C3	0.040 (2)	0.0448 (19)	0.036 (2)	−0.0013 (15)	0.0007 (16)	−0.0017 (16)
C4	0.045 (2)	0.052 (2)	0.035 (2)	0.0063 (17)	−0.0049 (16)	−0.0100 (17)
C5	0.041 (2)	0.045 (2)	0.044 (2)	0.0033 (15)	−0.0056 (17)	−0.0135 (17)
C6	0.0305 (19)	0.0419 (19)	0.042 (2)	−0.0021 (14)	−0.0051 (16)	−0.0079 (16)
C7	0.0276 (18)	0.0376 (18)	0.038 (2)	−0.0025 (14)	−0.0015 (15)	−0.0087 (15)
C8	0.0322 (19)	0.0325 (17)	0.038 (2)	−0.0014 (14)	0.0022 (15)	−0.0047 (15)
C9	0.0347 (19)	0.0286 (16)	0.0363 (19)	−0.0027 (14)	−0.0040 (15)	−0.0049 (14)
C10	0.0366 (19)	0.0342 (17)	0.0281 (18)	−0.0083 (14)	−0.0014 (14)	−0.0025 (14)
C11	0.041 (2)	0.0368 (18)	0.0319 (19)	−0.0070 (15)	−0.0025 (16)	−0.0067 (14)
C12	0.046 (2)	0.0345 (18)	0.039 (2)	−0.0126 (15)	−0.0011 (16)	−0.0058 (15)
C13	0.0367 (19)	0.0419 (19)	0.0368 (19)	−0.0120 (15)	0.0001 (15)	−0.0018 (15)
C14	0.041 (2)	0.0347 (17)	0.0348 (19)	−0.0082 (14)	0.0050 (16)	−0.0060 (14)
C15	0.0349 (19)	0.0332 (17)	0.0281 (18)	−0.0083 (14)	−0.0031 (14)	−0.0045 (14)
C16	0.038 (2)	0.0363 (18)	0.0263 (17)	−0.0087 (15)	−0.0010 (15)	−0.0074 (14)
C17	0.039 (2)	0.0368 (18)	0.0291 (18)	−0.0076 (15)	0.0006 (15)	−0.0113 (15)
C18	0.040 (2)	0.0302 (17)	0.0332 (19)	−0.0080 (14)	0.0021 (15)	−0.0083 (14)
C19	0.041 (2)	0.0389 (19)	0.042 (2)	−0.0123 (15)	0.0013 (16)	−0.0111 (16)
C20	0.037 (2)	0.0354 (18)	0.051 (2)	−0.0053 (14)	−0.0063 (17)	−0.0103 (16)
C21	0.044 (2)	0.0346 (18)	0.041 (2)	−0.0085 (16)	−0.0061 (17)	−0.0025 (15)
C22	0.0381 (19)	0.0377 (18)	0.0358 (19)	−0.0131 (15)	−0.0027 (15)	−0.0064 (15)
C23	0.0374 (19)	0.0281 (16)	0.0355 (19)	−0.0109 (14)	0.0002 (15)	−0.0083 (14)
C24	0.0335 (19)	0.0382 (18)	0.0300 (19)	−0.0081 (14)	0.0013 (15)	−0.0058 (15)
C25	0.0315 (18)	0.0347 (17)	0.037 (2)	−0.0046 (14)	0.0003 (15)	−0.0091 (15)
C26	0.040 (2)	0.0438 (18)	0.0281 (19)	−0.0090 (15)	0.0039 (15)	−0.0059 (14)
C27	0.037 (2)	0.0471 (19)	0.034 (2)	−0.0037 (15)	−0.0004 (16)	−0.0081 (16)
C28	0.0323 (18)	0.0350 (17)	0.036 (2)	−0.0040 (14)	−0.0002 (15)	−0.0113 (15)
C29	0.040 (2)	0.0316 (17)	0.036 (2)	−0.0057 (14)	0.0064 (15)	−0.0053 (14)
C30	0.044 (2)	0.0359 (17)	0.0294 (18)	−0.0081 (15)	−0.0008 (15)	−0.0042 (15)
C31	0.040 (2)	0.0415 (19)	0.043 (2)	−0.0057 (15)	0.0073 (17)	−0.0145 (16)
C32	0.063 (3)	0.066 (2)	0.058 (3)	−0.0208 (19)	−0.015 (2)	−0.019 (2)
B1	0.033 (2)	0.037 (2)	0.037 (2)	−0.0075 (17)	0.0000 (18)	−0.0036 (17)

### Geometric parameters (Å, °)

**Table:**

O1—C25	1.361 (3)	C12—C13	1.394 (4)
O2—C31	1.229 (3)	C12—H12A	0.9500
N1—C8	1.367 (3)	C13—C14	1.377 (4)
N1—C1	1.373 (3)	C13—H13A	0.9500
B1—O1	1.457 (4)	C14—C15	1.386 (4)
B1—N1	1.487 (4)	C14—H14A	0.9500
B1—N3	1.494 (4)	C15—C16	1.458 (4)
B1—N5	1.487 (4)	C17—C18	1.449 (4)
N2—C9	1.342 (4)	C18—C19	1.393 (4)
N2—C8	1.351 (3)	C18—C23	1.418 (4)
N3—C16	1.364 (4)	C19—C20	1.385 (4)
N3—C9	1.368 (3)	C19—H19A	0.9500
N4—C16	1.346 (3)	C20—C21	1.402 (4)
N4—C17	1.354 (3)	C20—H20A	0.9500
N5—C17	1.371 (4)	C21—C22	1.383 (4)
N5—C24	1.376 (3)	C21—H21A	0.9500
N6—C24	1.343 (4)	C22—C23	1.396 (4)
N6—C1	1.353 (3)	C22—H22A	0.9500
C1—C2	1.454 (4)	C23—C24	1.457 (4)
C2—C3	1.392 (4)	C25—C26	1.388 (4)
C2—C7	1.413 (4)	C25—C30	1.391 (4)
C3—C4	1.388 (4)	C26—C27	1.378 (4)
C3—H3A	0.9500	C26—H26A	0.9500
C4—C5	1.393 (4)	C27—C28	1.390 (4)
C4—H4A	0.9500	C27—H27A	0.9500
C5—C6	1.379 (4)	C28—C29	1.390 (4)
C5—H5A	0.9500	C28—C31	1.495 (4)
C6—C7	1.393 (4)	C29—C30	1.386 (4)
C6—H6A	0.9500	C29—H29A	0.9500
C7—C8	1.447 (4)	C30—H30A	0.9500
C9—C10	1.458 (4)	C31—C32	1.490 (5)
C10—C11	1.395 (4)	C32—H32A	0.9800
C10—C15	1.412 (4)	C32—H32B	0.9800
C11—C12	1.381 (4)	C32—H32C	0.9800
C11—H11A	0.9500		
			
C25—O1—B1	130.3 (2)	N4—C16—C15	130.5 (3)
C8—N1—C1	112.4 (2)	N3—C16—C15	105.9 (2)
C8—N1—B1	122.7 (2)	N4—C17—N5	122.4 (2)
C1—N1—B1	123.5 (2)	N4—C17—C18	130.3 (3)
C9—N2—C8	116.7 (2)	N5—C17—C18	106.1 (2)
C16—N3—C9	113.1 (2)	C19—C18—C23	120.4 (3)
C16—N3—B1	123.2 (2)	C19—C18—C17	132.4 (3)
C9—N3—B1	123.1 (3)	C23—C18—C17	107.1 (3)
C16—N4—C17	116.8 (3)	C20—C19—C18	118.1 (3)
C17—N5—C24	112.3 (2)	C20—C19—H19A	121.0
C17—N5—B1	123.0 (2)	C18—C19—H19A	121.0
C24—N5—B1	123.1 (3)	C19—C20—C21	121.2 (3)
C24—N6—C1	117.2 (2)	C19—C20—H20A	119.4
N6—C1—N1	121.8 (3)	C21—C20—H20A	119.4
N6—C1—C2	130.9 (3)	C22—C21—C20	121.6 (3)
N1—C1—C2	105.8 (2)	C22—C21—H21A	119.2
C3—C2—C7	120.6 (3)	C20—C21—H21A	119.2
C3—C2—C1	132.4 (3)	C21—C22—C23	117.4 (3)
C7—C2—C1	106.9 (3)	C21—C22—H22A	121.3
C4—C3—C2	117.7 (3)	C23—C22—H22A	121.3
C4—C3—H3A	121.2	C22—C23—C18	121.1 (3)
C2—C3—H3A	121.2	C22—C23—C24	131.3 (3)
C3—C4—C5	121.6 (3)	C18—C23—C24	107.4 (2)
C3—C4—H4A	119.2	N6—C24—N5	122.5 (3)
C5—C4—H4A	119.2	N6—C24—C23	130.7 (3)
C6—C5—C4	121.2 (3)	N5—C24—C23	105.4 (3)
C6—C5—H5A	119.4	O1—C25—C26	115.6 (3)
C4—C5—H5A	119.4	O1—C25—C30	124.9 (3)
C5—C6—C7	118.0 (3)	C26—C25—C30	119.4 (3)
C5—C6—H6A	121.0	C27—C26—C25	120.6 (3)
C7—C6—H6A	121.0	C27—C26—H26A	119.7
C6—C7—C2	120.8 (3)	C25—C26—H26A	119.7
C6—C7—C8	131.3 (3)	C26—C27—C28	120.6 (3)
C2—C7—C8	107.8 (3)	C26—C27—H27A	119.7
N2—C8—N1	122.8 (3)	C28—C27—H27A	119.7
N2—C8—C7	129.5 (3)	C27—C28—C29	118.5 (3)
N1—C8—C7	105.8 (2)	C27—C28—C31	122.0 (3)
N2—C9—N3	122.2 (3)	C29—C28—C31	119.5 (3)
N2—C9—C10	131.0 (3)	C30—C29—C28	121.4 (3)
N3—C9—C10	105.3 (3)	C30—C29—H29A	119.3
C11—C10—C15	120.6 (3)	C28—C29—H29A	119.3
C11—C10—C9	131.6 (3)	C29—C30—C25	119.5 (3)
C15—C10—C9	107.6 (2)	C29—C30—H30A	120.3
C12—C11—C10	117.9 (3)	C25—C30—H30A	120.3
C12—C11—H11A	121.1	O2—C31—C32	120.7 (3)
C10—C11—H11A	121.1	O2—C31—C28	120.2 (3)
C11—C12—C13	121.2 (3)	C32—C31—C28	119.1 (3)
C11—C12—H12A	119.4	C31—C32—H32A	109.5
C13—C12—H12A	119.4	C31—C32—H32B	109.5
C14—C13—C12	121.5 (3)	H32A—C32—H32B	109.5
C14—C13—H13A	119.2	C31—C32—H32C	109.5
C12—C13—H13A	119.2	H32A—C32—H32C	109.5
C13—C14—C15	118.1 (3)	H32B—C32—H32C	109.5
C13—C14—H14A	120.9	O1—B1—N5	118.0 (3)
C15—C14—H14A	120.9	O1—B1—N1	117.1 (3)
C14—C15—C10	120.6 (2)	N5—B1—N1	104.7 (3)
C14—C15—C16	132.4 (3)	O1—B1—N3	107.7 (2)
C10—C15—C16	107.0 (3)	N5—B1—N3	104.0 (3)
N4—C16—N3	122.2 (2)	N1—B1—N3	103.8 (2)
